# Primary cardiac lymphoma presenting as heart failure and atrioventricular block: case report

**DOI:** 10.3389/fonc.2025.1635860

**Published:** 2025-10-24

**Authors:** Jiyi Liu, Fengzhi Sun, Sixun Jia, Shulong Zhang, Yu Wang

**Affiliations:** ^1^ Heart Center, Affiliated Zhongshan Hospital of Dalian University, Dalian, China; ^2^ Hematology Center, Affiliated Zhongshan Hospital of Dalian University, Dalian, China

**Keywords:** primary cardiac lymphoma, diffuse large B-cell lymphoma, atrioventricular block, physiological pacing, case report

## Abstract

Primary cardiac lymphoma (PCL) is an extremely rare and highly aggressive malignancy that typically presents with nonspecific cardiac symptoms. We report a case of primary cardiac diffuse large B-cell lymphoma (DLBCL) with initial manifestations of heart failure and third-degree atrioventricular block. The patient presented with progressive dyspnea, palpitations, and unintentional weight loss. Transthoracic echocardiography revealed a large right atrial mass. The patient first underwent surgical resection of the cardiac tumor, and pathological examination confirmed non-GCB subtype DLBCL. During fractionated R-CHOP chemotherapy, the patient experienced cardiac arrest, necessitating the urgent implantation of a temporary pacemaker. Subsequently, a permanent pacemaker was implanted using a physiological pacing strategy. After six cycles of chemotherapy and ongoing pacing support, the patient achieved complete remission, with no evidence of tumor recurrence during the follow-up period. This case suggests that for PCL patients presenting with heart failure and third-degree atrioventricular block, a combination of surgical resection, fractionated R-CHOP chemotherapy, and physiological pacing may contribute to a favorable outcome.

## 1 Introduction

Primary Cardiac Lymphoma (PCL) is an exceedingly rare malignancy, accounting for only 1-2% of all Primary Cardiac Tumors (PCT) (1). Among these, diffuse large B-cell lymphoma (DLBCL) is the most common histological subtype, constituting approximately 80% of PCL cases (2). The clinical presentation of PCL is often highly nonspecific, with common symptoms including cardiac tamponade, congestive heart failure, atrioventricular block, and various arrhythmias, leading to frequent misdiagnosis as other cardiac diseases (3, 4). Imaging modalities, such as echocardiography, cardiac magnetic resonance imaging (MRI), and computed tomography (CT), are crucial for initial screening and typically reveal space-occupying lesions predominantly in the right atrium. The definitive diagnosis relies on pathological examination, primarily through cytological analysis of pericardial effusion or endomyocardial biopsy (5).

Immune status plays a critical role in the pathogenesis of PCL. Immunocompromised individuals (such as those with HIV infection or organ transplant recipients) have a significantly increased risk of developing PCL (6, 7). However, PCL can also occur in immunocompetent individuals, suggesting the involvement of other, yet unidentified, pathogenetic mechanisms or risk factors beyond immunosuppression.

Chemotherapy is the mainstay of PCL treatment and is currently considered the most effective therapeutic modality, significantly improving patient survival rates (8). Commonly used chemotherapy regimens include cyclophosphamide, doxorubicin, vincristine, and prednisone. Some patients may also benefit from B cell-targeted therapies or immunotherapy (9). Although surgical resection has a limited independent impact on patient survival, it holds significant value in alleviating symptoms and improving the quality of life, particularly for patients with severe cardiac dysfunction caused by the tumor (8). When PCL is complicated by arrhythmias, management must address both lymphoma and arrhythmia. Specific management strategies for arrhythmias may involve appropriate pharmacological therapy based on the type of arrhythmia (e.g., bradycardia, atrioventricular block, or tachycardia) and, when necessary, electrophysiological interventions (10).

## 2 Case presentation

**Figure 1:**
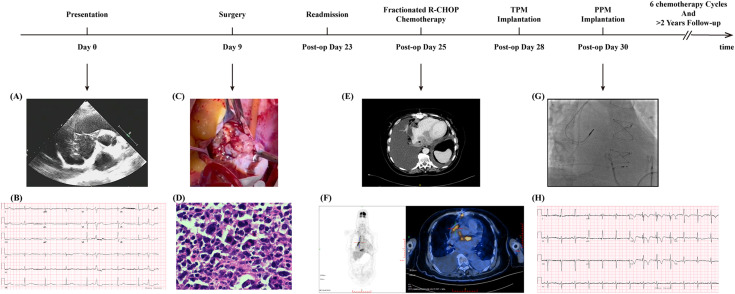
Timeline and representative clinical findings of the patient. A timeline illustrating the key clinical events, interventions, and findings during the patient’s diagnosis, treatment, and follow-up period. “Post−op” (post operation) refers to the period after surgical resection of the tumor. TPM, temporary pacemaker; PPM, Permanent Pacemaker. **(A)** Transthoracic echocardiography at initial presentation. A well-defined intracardiac mass involving the right atrium with dimensions of 62×39 mm. **(B)** Twelve-lead ECG showing complete atrioventricular block (third-degree AV block) with a junctional escape rhythm at approximately 50bpm. **(C)** Intraoperative photograph during tumor resection. A right atrial lobulated mass measuring approximately 70×45 mm, attached to the fossa ovalis of the interatrial septum, well circumscribed with a broad base, and nearly occupying the entire right atrium. **(D)** Histopathological examination confirmed lymphoma infiltration. Immunohistochemistry supported a diagnosis of diffuse large B−cell lymphoma, intermediate variant, classified as non−GCB subtype by the Hans algorithm. **(E)** Contrast-enhanced chest CT scan on the second admission revealed a residual mass. A soft tissue focus inferior to the atrium and a nodular lesion at the right cardiophrenic angle suggest possible lymphoma infiltration. Soft tissue is indicated by an asterisk. **(F)** PET-CT demonstrates significant metabolic activity of the cardiac tumor. Left: Whole-body maximum-intensity-projection (MIP) image showing focal intense uptake in the region of the interatrial septum. Right: Axial fused PET/CT image depicting interatrial septal thickening with adjacent dilation of the inferior vena cava, demonstrating intense radiotracer uptake on PET/CT (SUVmax 18.4); delayed imaging shows further increased uptake (SUVmax 21.8). **(G)** Pacemaker implantation displayed by fluoroscopy. The placement of the dual−chamber system and final lead position. Pacing parameters: Atrial lead 4574 (single/dual) positioned in the right atrial appendage; threshold 1.0/1.0 V; sensing 3.0/1.6 mV; impedance 840/740 Ω; Ventricular lead 3830 (single/dual) positioned at the right ventricular septum; threshold 0.9/0.7 V; sensing: temporary pacer capture; impedance 840/840 Ω. **(H)** ECG after pacemaker implantation. Heart rate 75 bpm with atrial sensing and ventricular pacing; pacing spikes precede the QRS complexes. Lead V1 shows a Qrs morphology, QRS duration 110 ms, and R-wave peak time in V5/V6 of 60 ms.

A woman in her 70s presented to the emergency room with shortness of breath and heart palpitations for 2-month. She only had a history of unintentional weight loss. Echocardiography revealed a solid mass in the right atrium, prompting admission for cardiac surgical evaluation (**Figures 1A, B**). On the ninth day of admission, cardiac tumor resection was performed under temporary pacemaker support. Intraoperatively, the mass, measuring 70 × 45 mm, was observed to be large with a smooth and lobulated surface, located within the right atrium and attached to the fossa ovalis of the interatrial septum (**Figure 1C**). However, complete resection could not be accomplished. Immunohistochemistry of the tumor showed a B-cell phenotype with CD20 and CD19 positivity (focally strong), BCL6 positivity, MUM1 positivity, BCL2 positivity, and a high proliferative index with Ki−67 (+, >85%). The germinal center marker CD10 was negative, consistent with a non-GCB profile by the Hans algorithm. c-MYC (IHC) was positive in approximately 20% of tumor cells and p53 in approximately 30% of cells. T-cell and other lineage markers were negative (CD3, CD5, and CD30), as were Cyclin D1, CD21, and CD34. Epithelial and myogenic markers were negative (AE1/AE3, Vimentin, Desmin, MyoD1, Myogenin). EBV−EBER *in situ* hybridization was negative. Overall, the findings supported a diagnosis of diffuse large B−cell lymphoma, intermediate variant, non−GCB subtype, according to the Hans classification (**Figure 1D**).

Twenty-three days postoperatively, the patient was hospitalized in the hematology department due to persistent fever, shortness of breath, and paroxysmal nocturnal dyspnea (PND) for 2-day. Physical examination revealed bradycardia, diminished breath sounds in both lower lung fields, and mild edema of both lower extremities. Laboratory tests showed a troponin I level of 0.166 ng/mL (reference range <0.001 ng/mL; conversion between ng/mL and μg/L is 1:1) and an N-terminal pro–brain natriuretic peptide (NT-proBNP) level of 1713 ng/L (reference range <125 ng/L). ECG showed complete heart block at a rate of 50 beats/min. Transthoracic echocardiography did not reveal any atrial masses. Contrast-enhanced chest CT revealed a large bilateral pleural effusion, a soft tissue lesion below the atrium, and a nodular lesion at the right cardiophrenic angle (**Figure 1E**). Positron emission tomography (PET) revealed hypermetabolic foci within the right atrium, interatrial septum, and adjacent inferior vena cava, with a maximum standardized uptake value (SUV) of 21.8 (**Figure 1F**). On postoperative day 28, during fractionated R-CHOP chemotherapy (on the third day of rituximab administration), the patient experienced sudden syncope. Cardiac monitoring showed a flat line that lasted approximately ten seconds before the patient spontaneously regained consciousness. Electrocardiography (ECG) demonstrated complete heart block with a ventricular rate of 33 beats per minute (**Supplementary Figure 1**). An emergency temporary pacemaker was implanted.

## 3 Clinical question

Is the R-CHOP regimen suitable for this patient, or should the chemotherapy regimen be modified?

Is third-degree atrioventricular block reversible in this patient, and does the patient require permanent pacemaker implantation? If so, what is the preferred pacing mode?

## 4 Interpretation

### 4.1 Regimen selection: modify standard R−CHOP due to high cardiac risk

Given the patient’s marked conduction vulnerability and hemodynamic fragility, standard R-CHOP was not appropriate. Contemporary guidance supports anthracycline modification (e.g., non-pegylated liposomal doxorubicin or anthracycline-sparing regimens) and split/sequential dosing in high-risk cardiac settings (11). Accordingly, we used a fractionated, cardioprotective schedule to preserve efficacy while minimizing acute decompensation risk: Rituximab: 100 mg daily Days 1–16; Cyclophosphamide: 400 mg on Days 7 and 8; Vinorelbine: 30 mg on Day 8; Liposomal Doxorubicin: 20 mg on Days 7 and 8; Dexamethasone: 15 mg on Days 7–10. Following this regimen, the patient underwent six cycles of chemotherapy.

### 4.2 High−risk AV block in cardiac lymphoma: indication for PPM and physiologic pacing

PCL-induced AV block may improve after cytoreduction in some cases (12–16), but the extent and timing are unpredictable at baseline (17). In this patient, syncope with brief asystole in the setting of complete heart block conferred a high risk of recurrent asystole and sudden death; thus, a permanent pacemaker (PPM) was indicated to ensure continuous electrophysiologic protection. Given the patient’s concomitant heart failure and anticipated high pacing burden, physiologic pacing was prioritized after a comprehensive evaluation (**Figures 1G, H**). This strategy aligns more closely with the patient’s clinical profile and is expected to ensure effective pacing while optimizing electromechanical synchrony and improving the heart failure prognosis (18).

## 5 Discussion

Management of primary cardiac lymphoma can, in some patients, involve overlapping challenges of arrhythmia, hemodynamic fragility, and chemotherapy induction−phase risks. In our case, these factors converged to necessitate targeted trade-offs in pacing and chemotherapy strategies.

Arrhythmias are a well-recognized clinical manifestation of cardiac lymphoma, particularly in primary cardiac lymphoma (PCL). Reported types include atrioventricular block (first to third degree), atrial fibrillation, and sinus tachycardia (8, 19). Notably, complete atrioventricular block (AVB) can present as the initial manifestation of PCL (9); however, its reversibility remains a matter of debate. Some case reports have indicated that a minority of patients may experience recovery of conduction over weeks to months (13–16). However, in our case, follow-up revealed complete pacemaker dependence, suggesting that the AVB was nonreversible, which is consistent with other case report (20). According to prior reports, the recovery of conduction block often temporally coincides with marked tumor regression on imaging, suggesting that the reversibility of AVB primarily depends on the reduction in tumor burden. The potential mechanisms may include relief of direct compression or infiltration of the atrioventricular node/His–Purkinje system, resolution of tumor−associated inflammation and edema, and restoration of local electrophysiologic homeostasis (14–16). Conversely, when deep infiltration of the tumor leads to fibrosis, necrosis, and scar replacement, it causes permanent structural injury; in such settings, conduction may fail to recover despite tumor resection or chemotherapy-induced shrinkage (10, 21), resulting in persistent pacemaker dependence. Accordingly, in hemodynamically stable patients, short-term observation under close monitoring to assess chemotherapy response may allow a cautious delay in the decision for permanent pacemaker implantation; however, pacing should be prioritized to ensure safety in those at risk of syncope or hypoperfusion. Overall, the prognosis of conduction block appears to depend largely on the reversibility of the underlying pathology; however, mechanistic insights and prospective clinical evidence remain limited.

Although R-CHOP remains the standard regimen for diffuse large B-cell lymphoma, patients with intracardiac involvement and conduction instability are at a heightened risk during induction. Early immunochemotherapy can impose significant physiological stress that aggravates arrhythmic vulnerability, and anthracyclines add dose−dependent cardiotoxicity, especially in individuals prone to decompensation (22). Cardio-oncology statements support anthracycline modification (e.g., non-pegylated liposomal doxorubicin or anthracycline-sparing regimens such as R-CEOP), along with split or sequential dosing and enhanced monitoring to attenuate acute cardiac events in high-risk settings (11). Consistent with these principles, we implemented a fractionated, cardioprotective schedule, split rituximab exposure, cyclophosphamide, vinca, and nonpegylated liposomal doxorubicin to balance cytotoxic efficacy with hemodynamic safety. The Adams–Stokes episode on day 3 of rituximab, given the current lack of definitive evidence that rituximab can directly cause cardiac arrest, is more plausibly attributable to local edema and inflammation from rapid tumor necrosis superimposed on an already fragile conduction system rather than a direct drug effect. Following permanent pacing, therapy proceeded without further cardiovascular events, and the patient remained relapse−free at two years, supporting the safety and effectiveness of this tailored approach.

Given that the cardiac tumor in this patient was not completely resected, pacemaker implantation, especially the placement of a right atrial lead, carries a potential risk of tumor thrombus dislodgement. Therefore, a leadless pacemaker was considered a viable option for procedural planning. Leadless systems avoid a pocket and transvenous leads, thereby reducing the infection risk and eliminating potential lead–tumor contact; however, most currently available devices are single-chamber right ventricular systems and cannot provide atrioventricular synchrony, conduction system pacing (CSP), or cardiac resynchronization therapy (CRT) (23). Transvenous systems enable dual/tri-chamber support and CSP/CRT, which is particularly important for pacing-dependent patients who require synchrony or physiologic activation. In this case, pre-implant reassessment identified no significant intracavitary mass on echocardiography and no intracardiac FDG uptake on PET/CT, indicating a relatively low risk of tumor thrombus dislodgement. Given the patient’s complete AV block and the anticipated high pacing burden, a single−chamber leadless pacemaker could increase the risk of pacing−induced cardiomyopathy (PICM) (24). Therefore, a transvenous pacing system was selected. For a comparison of transvenous versus leadless systems, see **Supplementary Table 2**.

Transvenous systems enable physiologic pacing: conduction system pacing (CSP, His−bundle or left bundle branch area) recruits the native His–Purkinje network, producing near−physiologic ventricular activation with narrower QRS and better inter−/intraventricular synchrony (25). Compared with right ventricular apical pacing, CSP mitigates desynchrony and has been associated with lower rates of pacing−induced cardiomyopathy, fewer heart−failure hospitalizations, and favorable remodeling, with the greatest benefit in patients with a high pacing burden or existing/anticipated left ventricular dysfunction (18). Left bundle branch area pacing (LPPAP) often provides lower and more stable capture thresholds with robust sensing, and CRT remains an appropriate alternative or upgrade if CSP is not feasible or if ventricular function declines despite CSP (26). In this patient, characterized by complete AVB, pacing dependency, elevated NT-proBNP, pleural effusions, and paroxysmal nocturnal dyspnea, LBBAP aligned with both immediate stabilization and long-term protection against dyssynchrony-related remodeling. This is the first report of employing a physiological pacing strategy in a patient with PCL complicated by atrioventricular block. The two−year follow−up characterized by the absence of heart failure readmissions and functional improvement highlights an additional pacing option that broadens clinical decision-making for such high-risk patients.

## 6 Conclusion

In summary, chemotherapy regimens for patients with primary cardiac tumors should be individualized based on the patient’s specific circumstances. Particular caution is warranted in selecting chemotherapeutic protocols when the tumor involves the cardiac conduction system. For patients with primary cardiac lymphoma complicated by third-degree atrioventricular block, physiological pacing was associated with symptomatic improvement in heart failure in this case; however, its long-term efficacy and safety still require further clinical validation.

## Data Availability

The original contributions presented in the study are included in the article/**Supplementary Material**. Further inquiries can be directed to the corresponding author.
