# Supplementary material for: Primary cardiac lymphoma presenting as heart failure and atrioventricular block: case report
Source: Front Oncol. 2025 Oct 24;15:1635860. doi: 10.3389/fonc.2025.1635860 (PMC12591941; doi:10.3389/fonc.2025.1635860)
Supplement: Supplementary file 1 [file DataSheet1.pdf]

Supplementary Material

1. Supplementary Figures

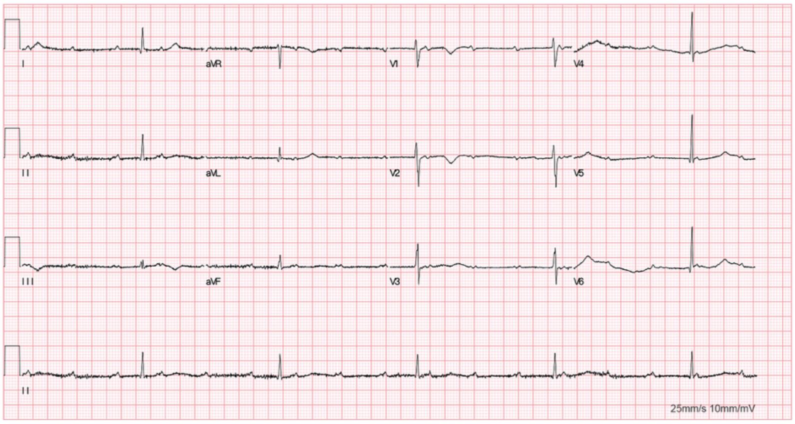

Supplementary Figure 1. Twelve-lead ECG before TPM implantation.
The ECG shows a complete atrioventricular block (third-degree AV block) with a junctional escape rhythm at approximately 33 bpm. TPM, temporary pacemaker.

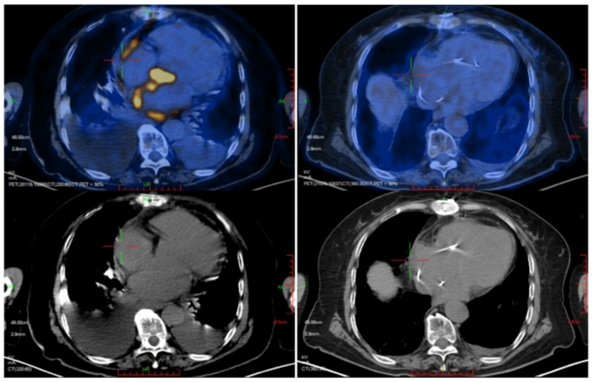

Supplementary Figure 2. PET/CT Findings on Follow-up (August 16, 2022)
A PET/CT scan performed on August 16, 2022, during follow-up, showed a localized mild increase in radiotracer uptake at the left pericardial wall (SUVmax: 3.5), which was significantly decreased

compared to the previous scan, suggesting reduced metabolic activity at this site. No evidence of tumor recurrence or distant metastasis was observed in any other organs or tissues.

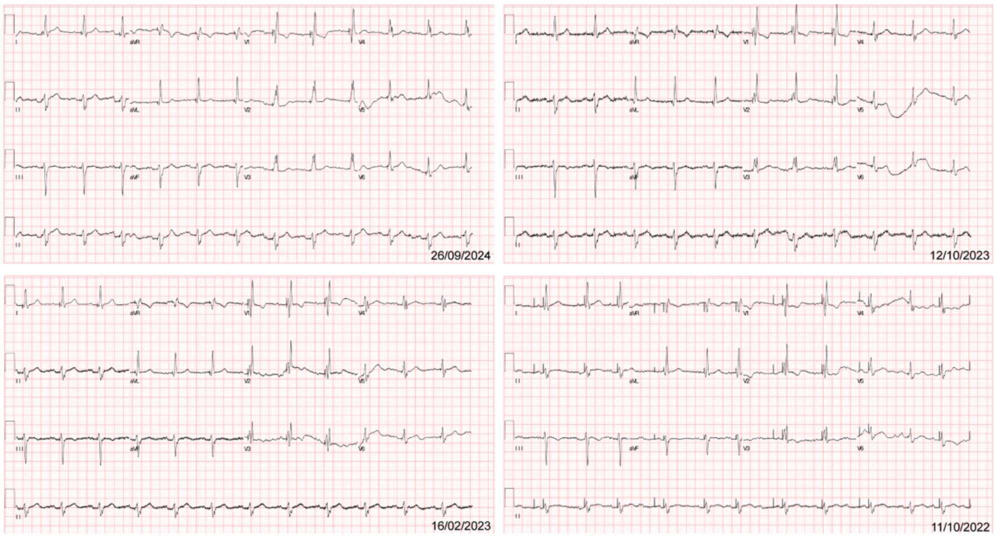

Supplementary Figure 3. Serial Electrocardiograms During Follow-up
Serial 12-lead electrocardiograms (ECGs) obtained during follow-up demonstrated complete ventricular pacing, indicating 100% pacemaker dependency throughout the observation period.

2. **Supplementary Table**

Examination Date	Nt-proBNP (pg/ml)	LVEF (%)	NYHA functional class	LVEDD (mm)	LVESD (mm)	IVS (mm)	LA Diameter (mm)	Mitral E Velocity (cm/s)	Mitral A Velocity (cm/s)	E/A Ratio
26/09/2024 Post-op Day 796	419	58	I	46	26	9	41	51	61	E<A
12/10/2023 Post-op Day 446	327	63	II	45	26	10	44	55	72	E<A
16/02/2023 Post-op Day 208	476	62	II	48	28	8	43	85	70	E>A

11/10/2022<br><br>Post-op Day 80	383	56	II	48	28	10	43	91	55	E>A
16/08/2022<br><br>Post-op Day 24	5369	68	IV	48	27	10	45	100	45	E>A

Supplementary Table 1. Serial Cardiac Function and NT-proBNP Measurements During Follow-up
Post-operation (post-op) refers to the period after surgical resection of the tumor. LVEF, left ventricular ejection fraction; NYHA, New York Heart Association; LVEDD, left ventricular end-diastolic diameter; LVESD, left ventricular end-systolic diameter; IVS, interventricular septal; LA, left atrial.

**Domain**	**Leadless Pacemaker (LP: single- and dual-chamber/AV-synchronous)**	**Transvenous Physiologic Pacing (TV-Physio: HBP/LBBAP ± dual chamber)**	**Conventional Transvenous RV Pacing (TV-RV: single-/dual-chamber; ± CRT)**	**Key Points in Oncology Patients**
System configuration	Intracardiac device(s), no pocket, no transvenous leads via SVC; single-unit RV (single-chamber) or two modules (RA + RV)	Subcutaneous generator + transvenous lead(s) targeting His bundle or left bundle branch area	Subcutaneous generator + RV lead (± RA lead); CRT requires a CS–LV lead	LP avoids pocket/lead issues, advantageous for infection control and radiotherapy planning
AV/ventricular synchrony	Single-chamber is typically VVI(R) (1); dual-chamber LP systems provide AV synchrony (DDD(R)-	Preserves AV and intraventricular synchrony; LBBAP typically offers more stable thresholds than HBP(4)	Dual-chamber can provide AV synchrony; isolated	For sustained AV/intraventricular synchrony needs, prioritize TV-Physio or CRT;

	equivalent)(2); CRT not available(3)		RV pacing is non-physiologic; CRT restores inter-ventricular synchrony when indicated(5)	dual-chamber LP is a valuable alternative when TV systems are unfavorable
Hemodynamics	Dual-chamber LP outperforms single-chamber LP and isolated RV pacing(6); inferior to CSP or CRT(7); single-chamber high RV pacing burden raises PICM risk(8)	Best among the three; lowers dyssynchrony/PICM and improves LVEF vs RV pacing(4)	Isolated RV pacing increases dyssynchrony and PICM risk(9); CRT counteracts dyssynchrony in appropriate candidates (10)	In heart failure or reduced LVEF, prioritize TV-Physio or CRT; dual-chamber LP is a secondary option when constraints exist
Venous access/thrombosis	Femoral–IVC approach; does not consume SVC access; negligible lead-related SVC thrombosis(11)	Requires SVC access; risk of lead-related venous stenosis/thrombosis (12)	Similar to TV-Physio; CRT carries a higher risk of thrombosis (13)	LP preferred in SVC stenosis/occlusion or high thrombotic risk
Infection risk	Lowest risk: no pocket/leads; device infection during bacteremia is rare(14)	Pocket/lead infection risk present; higher risk during neutropenia/chemotherapy(15)	Similar to TV-Physio	In neutropenia or immunosuppression, LP reduces infection-

				related complications
Radiotherapy	No chest pocket, reducing interference with radiation fields/dose; (16,17); Evidence on dual-chamber LP and radiotherapy is lacking	Leads/pocket may intersect target volumes, constraining field design and dose; radiotherapy may affect pacemaker function(18)	Similar to TV-Physio	LP facilitates radiotherapy planning; all strategies require imaging guidance when masses are present
Operative time	Small risks of perforation/tamponade(14); dual-chamber LP takes longer than single-chamber(6)	HBP requires precise mapping and threshold optimization(19); LBBAP is generally more reproducible(20)	Similar to TV-Physio; CRT longest(21)	When perioperative windows are tight or rapid stabilization is needed, LP is efficient; complex physiologic aims may favor TV strategies
Anticoagulation/bleeding	No pocket hematoma; femoral site bleeding usually minor; favorable with ongoing anticoagulation or thrombocytopenia(3)	Pocket hematoma risk(22); meticulous perioperative antithrombotic management required(23)	Similar to TV-Physio	LP is safer when bleeding risk is high or continuous anticoagulation is required
Longevity and replacement	~10–15 years typical(24,25); replacements often by stacking a new LP (late extraction is difficult); dual-chamber LP requires separate battery management for two devices	Generator ~8–15 years; generator exchange is straightforward(4)	Similar to TV-Physio; CRT longevity shorter due to higher output(26)	For younger/long-term survivors, plan for cumulative device burden from the outset

Upgrade pathways	Single-chamber LP can be complemented by a second LP for AV synchrony(27,28) ; overall no CRT/CSP capability; future upgrades often require switching to TV systems(28)	Highly flexible: add RA lead, upgrade to CRT, or change CSP site(29)	Upgradable to dual-chamber or CRT(30)	If future CRT/CSP is likely, prioritize a TV pathway
Cost and access	Device costs generally higher for LP (dual-chamber higher than single); access varies by region(31)	Moderate device costs; dependent on operator expertise and tools(32)	Often more accessible; CRT cost is higher(33)	Cost-effectiveness hinges on preventing complications and achieving hemodynamic benefit
Ideal candidates	High infection/bleeding risk; SVC access limitations; need for chest radiotherapy; if AV synchrony required, consider dual-chamber LP	High pacing burden; HF or reduced LVEF; need for physiologic activation or potential CRT in future	Intermittent pacing needs; good venous access; clear CRT indications with suitable CS anatomy	Individualize based on access, infection/thrombotic risk, pacing burden, HF phenotype, and upgrade needs

Supplementary Table 2. Key considerations for Leadless vs. Transvenous Physiological vs. Transvenous Traditional RV Pacing in Oncology-Related Conduction Disease
LP, leadless pacemaker; TV-Physio, transvenous physiologic pacing; TV-RV, conventional transvenous right ventricular pacing; CRT, cardiac resynchronization therapy; PICM, pacing-induced cardiomyopathy; SVC, superior vena cava; CS-LV, coronary sinus–left ventricular lead; AV, atrioventricular; LVEF, left ventricular ejection fraction.

Reference

1. Gurgu A, Petrescu L, Luca CT, Văcărescu C, Târtea G, Goanță E-V, Cirin L, Cozma D. Update in LV only fusion CRT pacing: annals and future perspectives. *Curr Health Sci J* (2023) 49:479–486. doi: 10.12865/CHSJ.49.04.01

2. Ip JE, Rashtian M, Exner DV, Reddy VY, Doshi R, Badie N, Nevo JR, Goil A, Defaye P, Canby R, et al. Atrioventricular synchrony delivered by a dual-chamber leadless pacemaker system. *Circulation* (2024) 150:439–450. doi: 10.1161/CIRCULATIONAHA.124.069006

3. Saleem-Talib S, Hoevenaars CPR, Molitor N, van Driel VJ, van der Heijden J, Breitenstein A, van Wessel H, van Schie MS, de Groot NMS, Ramanna H. Leadless pacing: a comprehensive review. *Eur Heart J* (2025) 46:1979–1990. doi: 10.1093/eurheartj/ehaf119

4. Whinnett Z, Naraen A, Vijayaraman P, Cleland JGF, Keene D. Physiological pacing: mechanisms, clinical indications, and perspectives. *Eur Heart J* (2025) 46:3407–3419. doi: 10.1093/eurheartj/ehaf440

5. Das A, Kahali D. Physiological cardiac pacing: current status. *Indian Heart J* (2016) 68:552–558. doi: 10.1016/j.ihj.2016.03.033

6. Knops RE, Reddy VY, Ip JE, Doshi R, Exner DV, Defaye P, Canby R, Bongiorni MG, Shoda M, Hindricks G, et al. A dual-chamber leadless pacemaker. *N Engl J Med* (2023) 388:2360–2370. doi: 10.1056/NEJMoa2300080

7. Somani S, Rogers AJ. Advances in cardiac pacing with leadless pacemakers and conduction system pacing. *Curr Opin Cardiol* (2024) 39:1. doi: 10.1097/HCO.0000000000001092

8. Saeed Al-Asad K, Martinez A, Prasad RM, Ukponmwan EU, Baloch ZQ, Ali A, Ip J. Pacing-Induced Cardiomyopathy in Leadless and Traditional Pacemakers: A Single-Center Retrospective Analysis. *Cureus* (2023) doi: 10.7759/cureus.41393

9. Aritajati T, Tipcome K, Chitsomkasem A, Tokavanich N, Ananwattanasuk T, Teerawongsakul P. The prevalence and short-term outcomes of ventricular dyssynchrony after right ventricular pacing. *Int J Clin Med* (2021) 12:459–470. doi: 10.4236/ijcm.2021.1211042

10. Mechanical dyssynchrony and response to cardiac resynchronization therapy in heart failure patients with right ventricular pacing: a pre-specified subgroup analysis of the Budapest CRT upgrade trial | european heart journal | oxford academic. https://academic.oup.com/eurheartj/article/45/Supplement_1/ehae666.729/7838373 [Accessed October 3, 2025]

11. Soejima K, Hilpisch K, Samec ML, Temple RL, Bonner MD. Jugular approach for the transcatheter pacemaker implant ― better access for smaller hearts? ―. *Circ J* (2024) 88:1127–1134. doi: 10.1253/circj.CJ-24-0083

12. Safi M, Akbarzadeh MA, Azinfar A, Namazi MH, Khaheshi I. Upper extremity deep venous thrombosis and stenosis after implantation of pacemakers and defibrillators; a prospective study. *Rom J Intern Med = Rev Roum Med Interne* (2017) 55:139–144. doi: 10.1515/rjim-2017-0018

13. Shaik NA, Drucker M, Pierce C, Duray GZ, Gillett S, Miller C, Harrell C, Thomas G. Novel two-lead cardiac resynchronization therapy system provides equivalent CRT responses with less complications than a conventional three-lead system: results from the QP ExCELs lead registry. *J Cardiovasc Electrophysiol* (2020) 31:1784–1792. doi: 10.1111/jce.14552

14. Reynolds D, Duray GZ, Omar R, Soejima K, Neuzil P, Zhang S, Narasimhan C, Steinwender C, Brugada J, Lloyd M, et al. A leadless intracardiac transcatheter pacing system. *N Engl J Med* (2016) 374:533–541. doi: 10.1056/NEJMoa1511643

15. Goutam D. A study on pacemaker pocket infection. *J Cardiol Cardiovasc Med* (2020) 5:56–59. doi: 10.29328/journal.jccm.1001087

16. Ishihara S, Kashiwagi S, Asano Y, Tauchi Y, Morisaki T, Noda S, Takashima T, Onoda N, Hirakawa K, Ohira M. [a case of recurrent breast cancer treated with local irradiation using leadless pacemaker implantation]. *Gan Kagaku Ryoho, Cancer Chemother* (2019) https://www.semanticscholar.org/paper/%5BA-Case-of-Recurrent-Breast-Cancer-Treated-with-Ishihara-Kashiwagi/517653b96cf0effc0ae8991ab5e95a5f81dc05b9 [Accessed October 3, 2025]

17. Georges G, Philippon F, Champagne J, Albert E, O'Hara GE. Leadless pacemaker implantation in a patient with hypoplasia of the left brachiocephalic vein. *CJC Open* (2022) 4:810–812. doi: 10.1016/j.cjco.2022.05.005

18. Azraai M, Miura D, Lin Y-H, Rodrigues TS, Nadurata V. Incidence and predictors of cardiac implantable electronic devices malfunction with radiotherapy treatment. *J Clin Med* (2022) 11:6329. doi: 10.3390/jcm11216329

19. Imnadze G, Vijayaraman P, Bante H, Eitz T, Bergau L, Baridwan N, El Hamriti M, Molatta S, Braun M, Khalaph M, et al. Novel electroanatomical map for permanent his bundle pacing: the mont blanc approach - influence of the learning curve and procedural outcome. *EP Eur* (2020) 22:1697–1702. doi: 10.1093/europace/euaa226

20. Sharma PS, Vijayaraman P, Burri H. Complications, troubleshooting and follow-up for left bundle branch area pacing. *Arrhythmia Electrophysiol Rev* (2025) 14:e20. doi: 10.15420/aer.2024.12

21. Baroni M, Preda A, Carbonaro M, Fortuna M, Caccia A, Guarracini F, Colombo G, Vargiu S, Varrenti M, Frontera A, et al. LBBAP versus conventional CRT reimplantation after transvenous lead extraction: report from a monocentric experience. *Eur Heart J* (2024) 45:ehae666.728. doi: 10.1093/eurheartj/ehae666.728

22. Richter S, Gebauer R, Ebert M, Moscoso Ludueña C, Scheller D, Lucas J, König S, Paetsch I, Hindricks G, Döring M. Electroanatomical mapping–guided left bundle branch area pacing in patients with structural heart disease and advanced conduction abnormalities. *EP Eur* (2023) 25:1068–1076. doi: 10.1093/europace/euac232

23. Roseboom E, Smit MD, Groenveld HF, Rienstra M, Maass AH. Clinical decision making and technical approaches in implantable cardioverter-defibrillator procedures: a step by step critical appraisal of literature. *Rev Cardiovasc Med* (2024) 25:403. doi: 10.31083/j.rcm2511403

24. Breeman KTN, Oosterwerff EFJ, Dijkshoorn LA, Salavati A, Beurskens NEG, Wilde AAM, Delnoy P-PHM, Tjong FVY, Knops RE. Real-world long-term battery longevity of micra leadless pacemakers. *J Interventional Card Electrophysiol* (2022) 66:839–841. doi: 10.1007/s10840-022-01447-y

25. Certo Pereira J, Amador R, Reis Santos R, Bello R, A Gomes D, Rodrigues G, Matos D, Carmos J, Santos I, Galvao Santos P, et al. Leadless pacemaker - lead parameters stability and battery longevity in the mid-term follow-up. *Eur Heart J* (2024) 45:ehae666.700. doi: 10.1093/eurheartj/ehae666.700

26. Boriani G, Ritter P, Biffi M, Ziacchi M, Diemberger I, Martignani C, Valzania C, Valsecchi S, Padeletti L, Gadler F. Battery drain in daily practice and medium-term projections on longevity of cardioverter-defibrillators: an analysis from a remote monitoring database. *Europace* (2016) 18:1366–1373. doi: 10.1093/europace/euv436

27. Clementy N, Chinitz J, Marijon E, Haeberlin A, Winter S, Iacopino S, Curnis A, Breitenstein A, Hussin A, Mela T, et al. A leadless ventricular pacemaker providing atrioventricular synchronous pacing in the real-world setting: acute results from the micra AV post-approval registry. *Europace* (2023) 25:euad122.392. doi: 10.1093/europace/euad122.392

28. Stazi F. Leadless pacemaker 5-year outcomes: good news? *Eur Heart J Suppl* (2025) 27:iii150–iii152. doi: 10.1093/eurheartjsupp/suaf034

29. Merkely B, Kosztin A, Roka A, Geller L, Zima E, Kovacs A, Boros AM, Klein H, Wranicz JK, Hindricks G, et al. Rationale and design of the Budapest-CRT upgrade study: a prospective, randomized, multicentre clinical trial. *Europace* (2016)euw193. doi: 10.1093/europace/euw193

30. Bongiorni MG, Reddy VY, Ip JE, Doshi R, Exner DV, Defaye P, Canby R, Shoda M, Hindricks G, Rashtian M, et al. Upgrading a single-chamber leadless pacemaker to a dual-chamber leadless pacemaker system. *Europace* (2024) 26:euae102.392. doi: 10.1093/europace/euae102.392

31. Makino K, Mudge M, Hill M, Zaunmayr C, Tilden D. Cost-effectiveness of micra^{TM} VR leadless pacemaker in patients with bradycardia and atrial fibrillation in Australia. *J Arrhythmia* (2024) 40:1481–1489. doi: 10.1002/joa3.13145

32. Chaumont C, Auquier N, Milhem A, Mirolo A, Al Arnaout A, Popescu E, Viart G, Godin B, Gillibert A, Savouré A, et al. Can permanent his bundle pacing be safely started by operators new to this technique? Data from a multicenter registry. *J Cardiovasc Electrophysiol* (2021) 32:417–427. doi: 10.1111/jce.14860

33. Landolina M, Morani G, Curnis A, Vado A, D’Onofrio A, Bianchi V, Stabile G, Crosato M, Petracci B, Ceriotti C, et al. The economic impact of battery longevity in implantable cardioverter-defibrillators for cardiac resynchronization therapy: the hospital and healthcare system perspectives. *EP Eur* (2017) 19:1349–1356. doi: 10.1093/europace/euw176
